# EM-YOLO: An X-ray Prohibited-Item-Detection Method Based on Edge and Material Information Fusion

**DOI:** 10.3390/s23208555

**Published:** 2023-10-18

**Authors:** Bing Jing, Pianzhang Duan, Lu Chen, Yanhui Du

**Affiliations:** 1 School of Information and Network Security, People’s Public Security University of China, Beijing 102206, China; ice.bingjing@foxmail.com; 2 School of Information Engineering, Shenyang University of Chemical Technology, Shenyang 110142, China; pz_duan@163.com; 3 School of Vehicle and Mobility, Tsinghua University, Beijing 100190, China; chenlu0419@mail.tsinghua.edu.cn

**Keywords:** X-ray security inspection, deep learning, object detection, attention mechanism

## Abstract

Using X-ray imaging in security inspections is common for the detection of objects. X-ray security images have strong texture and RGB features as well as the characteristics of background clutter and object overlap, which makes X-ray imaging very different from other real-world imaging methods. To better detect prohibited items in security X-ray images with these characteristics, we propose EM-YOLOv7, which is composed of both an edge feature extractor (EFE) and a material feature extractor (MFE). We used the Soft-WIoU NMS method to solve the problem of object overlap. To better extract features, the attention mechanism CBAM was added to the backbone. According to the results of several experiments on the SIXray dataset, our EM-YOLOv7 method can better complete prohibited-item-detection tasks during security inspection with detection accuracy that is 4% and 0.9% higher than that of YOLOv5 and YOLOv7, respectively, and other SOTA models.

## 1. Introduction

X-ray security inspection is important for public safety, and imaging benefits from perspective characteristics. X-ray imaging is used to scan luggage and find prohibited items hidden in luggage [1]. Many accidents are caused by unsafe human behaviour. X-ray images are becoming increasingly indispensable for security purposes [2]. With the rapid development of artificial intelligence, the implementation of intelligent security checks via machine-assisted artificial labour is of great significance in improving the work efficiency of security inspectors. A detection algorithm is used with X-ray imaging to determine whether prohibited items are present and identify, classify, and mark their position in the image. X-ray inspection images have the following characteristics. (1) Background clutter [3,4]: due to the correlation between the colour and material of the X-ray image, when the thickness and density of the prohibited items is similar to the background, the background will interfere with feature learning of the prohibited items [5]. (2) Object overlap [6,7,8,9]: the shape of an object is seriously distorted under ray projection, and random placement will lead to occlusion between objects, which will increase the difficulty of prohibited item identification [5]. Therefore, detecting the background interference and overlapping occlusion of prohibited items presents a challenge in X-ray image detection.

In recent years, object detection has undergone continuous development from simple images. Many researchers have improved the existing object detection network structure for different task scenarios. However, due to the characteristics of X-ray images, feature extraction networks designed for traditional images have poor adaptability, and improving the network is necessary. For example, some researchers have utilized an attention mechanism to achieve more accurate feature extraction [10,11,12,13,14,15]. For objects with significant size differences, a multiscale feature fusion method has been proposed [12,16,17,18,19]. However, there is no network that can effectively solve the problems of object overlap and background clutter. To address these issues, we were inspired by the design concept of DOAM [14] and referred to its optimized edge detection (EG) and material awareness (MA) module. We designed an edge feature extractor (EFE) and material feature extractor (MFE) to better extract features from X-ray images. To address the issue of overlapping occlusion, we used the Soft-WIoU NMS method [20,21] to process the detection results.

Our contribution can be summarized as follows:

(1) We designed an EM attention module to address the complex background of X-ray inspection images. Feature extraction from the attention area formed by edge feature fusion of the material RGB feature can quickly and accurately allow prohibited item detection.
(2) We proposed a Soft-NMS based on WIoU loss function to solve the problem of object overlap and achieved good results. Compared to the original NMS, Soft-NMS places more emphasis on the selection of prediction boxes with overlapping positions and includes a WIoU penalty term to improve accuracy.
(3) To better extract features, we added CBAM [22] to the backbone network for comparison with other attention mechanisms.

## 2. Related Works

### 2.1. Prohibited Item Detection in X-ray Images

X-rays are widely used for security inspection, such as in train stations, airports, and subway stations. X-rays have strong penetration ability, but due to scene problems, the detected objects are stacked and occlude each other, there is substantial background noise, and prohibited items share many characteristics with non-prohibited items. To better complete security inspection tasks, a large number of studies have been devoted to detecting prohibited items in X-ray inspection images. Due to the advanced nature of current deep learning technology, an increasing number of researchers have been using methods based on convolutional neural networks and have been improving upon them to solve existing problems, such as YOLO [23] and SSD [24]. The TB-YOLOv5 [11] network uses the attention-BiFPN attention mechanism to enhance the features, which has improved the detection accuracy of small objects. M-SSD [25] handles detection problems in cluttered backgrounds better by incorporating feature fusion modules and asymmetric convolutions. Zhou et al. [6] used Soft-NMS to optimize stacked detection. Hong Duc Nguyen et al. [26] proposed a task-driven cropping scheme, called TDC, to crop out redundant backgrounds in X-ray images. Wei et al. proposed a method to synthesize X-rays, which effectively increased the number of positive samples in the dataset, and proposed a mask RCNN based on Softer-NMS. Zhou’s model [6], an improvement of YOLOv4 [27], explores the overlap problem by defining a new loss function.

As research has progressed, an increasing number of datasets have appeared in this field in recent years. The GDXray [28] dataset includes 19,407 samples with multiple views, but greyscale images are not suitable for current security check scenarios. The OPIXray [14] dataset is used to detect sharp objects and sets three different levels of occlusion for training and validation. The SIXray [29] dataset is a dataset which is commonly used in research that imitates the real-world situation where the positive sample ratio is very small, with balanced categories that cover common prohibited items. This paper mainly studies solutions to the problem of a large number of stacked objects.

### 2.2. Attention Mechanism

Adding different attention mechanisms to the same network for different detection tasks has been effective. The attention mechanism refers to the allocation of available computing resources to the parts of a feature that need to be detected, and this method has been widely studied for different types of tasks. DANet [30] proposes a dual attention network for performing scene segmentation tasks. By using a self-attention mechanism and capturing contextual dependencies, exclusive tasks can be completed. The squeeze and excitation network SENet [31] contains the squeeze and excitation block (SE), which adaptively recalibrates channel feature responses by explicitly modelling the interdependence between channels. The CBAM [22] attention mechanism is a simple and effective attention module for a feedforward convolutional neural network, which can be seamlessly integrated into any CNN architecture without considering cost. Unlike existing channel/spatial attention modules, SimAM [32] proposes deriving 3D attention weights for feature maps without the need for additional parameters. Most SimAM operations are based on a defined energy function selection, avoiding excessive structural adjustments.

There are also many studies that have used attention mechanisms in the field of X-ray detection for security purposes. Xu [15] utilized semantic information to form attention maps and better complete detection tasks. Zhao [8] used a label attention mechanism on the self-built dataset CLCxray to solve the overlap problem. Wu et al. [17] used multi-view primary and secondary channel attention filtering to effectively extract features from multi-view X-ray images. Song et al. [33] added the stem module to YOLOv5, which endowed the network with strong feature representation capabilities. Ren et al. [18] achieved good results using CBAM on the basis of YOLOv4 for small prohibited items. Zhang et al. [13] effectively extracted target object regions with distortion in feature maps using the malformed attention mechanism MAM. Viriyasaranon et al. [19] also used attention mechanisms in their proposed MFA-net. MCRPN [34] uses an attention mechanism to extract the corresponding feature maps from multiscale regions. SA-CenterNet [35] uses a feature enhancement module (FEM) to extract small and abstract object features.

Our model adds EM attention on top of YOlOv7 to better extract X-ray features, utilizes Soft NMS combined with WIoU to solve the problem of object overlap, and utilizes CBAM to enhance context connection and region of interest attention. In summary, the EM-YOLOv7 model has achieved good results in X-ray image detection.

## 3. EM-YOLOv7 Model

We propose using a special attention mechanism, edge and material attention (EM), on X-ray images, which uses the edge feature extractor (EFE) to extract the effective edge features in the X-ray image and uses the material feature extractor (MFE) to focus on the coloured areas of prohibited items to form an attention mechanism. We referred to the design concept of DOAM and considered its excessive and tedious feature extraction fusion process, ultimately simplifying its approach to form a new attention module (EA) designed specifically for X-ray images. Soft-NMS and WIoU are used in the network, and CBAM is added to the backbone. This chapter will introduce the network structure and the design of each module.

### 3.1. Base Model

**Figure 1:**
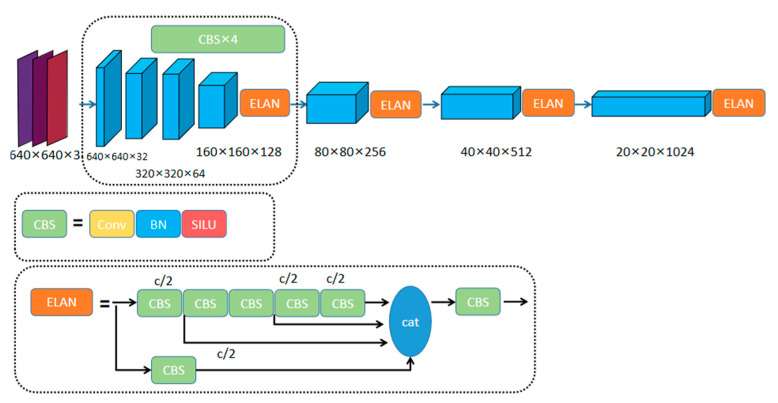
YOLOv7 deep learning network structure.

The basic network is the object detection network YOLOv7, which has been widely adopted by researchers for constantly developing object detection tasks. On this basis, feature extraction was inspired by DOAM. Many representative classification networks have been used for feature extraction, such as ResNet [36] and DenseNet [37]. Since YOLOv7 enables deeper networks to learn more effectively by controlling the shortest and longest paths, we use the YOLOv7 architecture. In the backbone module, we use both max pooling and convolution with a stride of two, both of which are the most commonly used methods. The input image first passes through three convolution modules. Then, the feature goes into three modules consisting of an ELAN module and downsampling module in sequence. Finally, the feature is enhanced by an ELAN module. The input image thus becomes a feature map. The details of the feature extractor are shown in Figure 1.

**Figure 2:**
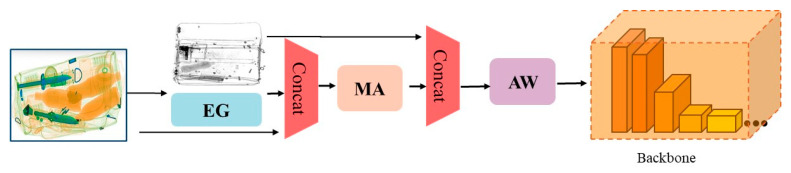
The pipeline of the DOAM.

The de-occlusion attention module (DOAM) is an attention mechanism used to solve occlusion problems. DOAM is placed on the front end of the SSD backbone to process the features in the X-ray images. The overall idea of DOAM is to concatenate the edge map generated by edge guidance (EG) and the original input image and then send them to attention generation (AG) for regional clustering. Then, the features of the two modules are fed into Conv to extract the features before the input backbone. Figure 2 shows the pipeline of the DOAM.

We consider that there is no better performance after the original input X concatenates the edge mask, and we also found that DOAM does not generate attention based on the material (RGB) of a prohibited item in the X-ray image. Therefore, we optimized the design and proposed EM-YOLOv7.

### 3.2. Network Architecture

**Figure 3:**
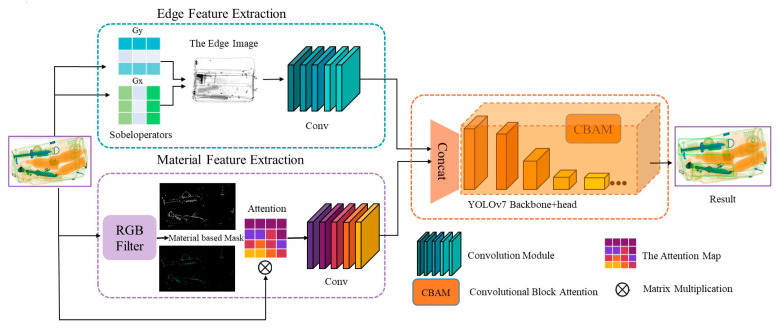
Schematic diagram of the EM-YOLOv7 deep learning network structure.

Figure 3 shows the network structure of EM-YOLOv7. On the basis of YOLOv7, we added our proposed EM attention module and used WIoU-trained Soft NMS in the prediction box when optimizing the downstream detection tasks. The EM attention module includes two feature extractors, namely, the edge feature extractor (EFE) and material feature extractor (MFE). After fusing these two features, the features are fed into the YOLOv7 network for subsequent feature extraction. In the YOLOv7 backbone, we add CBAM to each branch, so that the data are sent to the head to enhance the context connection and region of interest.

In the EFE module, the input image $X\in R^{C\times H\times W}$ is subjected to vertical and horizontal Sobel operators represented by convolution to obtain the feature map of the edge. Unlike DOAM, we do not overlap the edge map with the original image X. Instead, we use convolution to extract the feature $F_{E}\in R^{C\times H\times W}$, which is then concatenated with the feature $F_{M}\in R^{C\times H\times W}$ extracted by the material feature extractor. In the material feature extractor module, the same image $X\in R^{C\times H\times W}$ is input as the upper branch, and the mask of the material is obtained by the RGB filter manually set for prohibited items. On this basis, the generated attention distribution result is multiplied by the original input to extract the feature. This allows the model to focus more on the area of the prohibited items. It is worth mentioning that, in this step, there is a priori knowledge of the RGB of prohibited items in the $X\in R^{C\times H\times W}$ image.

In contrast, DOAM does not use RGB to form the attention area of prohibited items but uses complex pooling, concatenation, and other operations to enrich the extracted features. We think that this step is redundant, so after extracting the two parts of the features, we directly send them into the YOLOv7 network after size alignment, forming our network architecture.

### 3.3. Edge Feature Extraction (EFE)

The Sobel operator is a classic method for image edge detection. When edge detection is performed on an image, the gradient of each pixel is calculated, and the maximum change and rate of change from light to dark in different directions are given. This result indicates whether the change in brightness of the image at that point is “sharp” or “smooth”, which can determine the probability of the area becoming an edge. In practical operation, the possibility of being an edge is more reliable and convenient to calculate than the direction of calculation. At each pixel in the image, the gradient vector only considers the direction with the largest increase in brightness, and the length of the gradient vector corresponds to the rate of light intensity change in that direction. This means that the Sobel operator of a point in an area on the same pixel image is a zero vector, and a set of vector values on the edge line are brightness gradients. The Sobel operator’s process of image processing is essentially a continuous operation of difference and smoothing. Among them, [1, 0, −1] and its transposition represent the horizontal difference and vertical difference, respectively, whereas [1, 2, 1] and its transposition represent horizontal smoothing and vertical smoothing, respectively.

We applied 3 × 3 kernel size convolution to the original image to calculate the approximate gradient of changes in both the horizontal and vertical directions. We input image X as the horizontal and vertical approximate gradients of an image and calculate them as follows:

(1) $$G_{x}=\left[\begin{array}{ccc} -1 & 0 & +1\\ -2 & 0 & +2\\ -1 & 0 & +1 \end{array}\right]{\times X\;\;\;G}_{y}=\left[\begin{array}{ccc} -1 & -2 & -1\\ 0 & 0 & 0\\ +1 & +2 & +1 \end{array}\right]\times X$$

where × represents the convolution calculation. We combine the above two results $G_{x}\in R^{1\times H\times W}$ and $G_{y}\in R^{1\times H\times W}$ and further conclude that, to avoid background complexity, the extracted feature maps are sent to the 3 × 3 kernel size convolution module to further extract the edge map E. The convolution operation is the convolution of five layers of channel numbers, a batch normalization layer and an activation function layer to finally extract the edge feature $F_{E}$. The operations can be formulated as follows:

(2) $$F_{E}=\mathrm{ReLu}(X⊗E)$$

where ReLu represents the activation function.

### 3.4. Material Feature Extraction (MFE)

One of the characteristics of X-ray images is that metal materials have specific colours. We thus provide a new idea to generate attention channels: use prior knowledge of prohibited items to design a material feature extractor. First, input image X into the RGB filter. This filter will filter according to the RGB range of prohibited items to generate a material mask. Then, after the Softmax operation according to the mask generated by the filter, we can obtain the weight that is more inclined to the object to be detected, that is, the attention map. By multiplying the input image X and the attention map, we can extract the features that are more concerned with the area with prohibited items. Similar to the feature extraction module above, the map is composed of five convolution layers, one batch normalization layer, and one activation function. The specific formula is as follows:

(3) $$f_{M}=X⊗{(\mathrm{Softmax}(W}_{1}\cdot \mathrm{Filt}(X)))$$

(4) $${\mathrm{Softmax}(W}_{1}\times \mathrm{Filt}(X))=\frac{exp\left(W_{1}\times Filt\left(X\right)\right)}{\sum_{j}exp\left(x_{j}\right)}$$

(5) $$F_{M}=\mathrm{ReLu}(X⊗{\;f}_{M})$$

where $f_{M}\in R^{C\times H\times W}$ is the feature extracted from the attention channel, $W_{1}\in R^{H\times W}$ is the weight, and Filt is the RGB filter, which together result in the material feature $F_{M}$.

Our main goal in this step is to use the RGB filter to generate a mask and use the mask to generate an attention heatmap so that subsequent feature extraction will focus more on image areas with objects or even prohibited items.

### 3.5. Soft-WIoU-NMS

As the core problem of computer vision, object detection performance depends on the design of the loss function. The boundary box loss function is an important part of the target detection loss function and giving it good definition will bring a significant improvement to the performance of the object detection model. In recent years, most studies have assumed that the examples in the training data are high quality, aiming to enhance the fitting ability of boundary box losses. The previously existing IoU adds different penalties R on top of the existing penalties to adapt the IoU loss function to different problems. WIoU proposes a dynamic nonmonotonic focusing mechanism, which reduces the competitiveness of high-quality anchor frames while also reducing the harmful gradients generated by low-quality examples. This allows the WIoU to focus on ordinary quality anchor frames and improve the overall performance of the detector.

(6) $$L_{\mathrm{IoU}}=1-\mathrm{IoU}=1\;-\;{\frac{W_{i}H_{i}}{S_{u}}}_{\;}$$

(7) $$L_{i}\;{=L}_{\mathrm{IoU}}{\;+\;R}_{i}{\left(\mathrm{DIoU}/\mathrm{EIoU}/\ldots \right)}_{\;}{}_{\;}$$

(8) $$L_{\mathrm{WIoUv}1}{\;=R}_{\mathrm{WIoU}}L_{\mathrm{IoU}}{}_{\;}$$

(9) $$R_{WIoU}=exp{\left(\frac{{\left(x-x_{gt}\right)}^{2}+{\left(y-y_{gt}\right)}^{2}}{{\left(W_{g}^{2}+H_{g}^{2}\right)}^{*}}\right)}_{\;}{}_{\;}$$

Here,${\;W}_{I},I$ refers to the length and height of the prediction box, $L_{\mathrm{IoU}}$ is the original IoU definition, $I_{i}$ is the IoU improved paradigm, $l_{\mathrm{WIoUv}1}$ is the WIoUv1, and $r_{\mathrm{WIoU}}$ is the penalty for WIoU.

NMS is an algorithm designed to remove duplicate prediction boxes. The specific steps are as follows. Input all possible prediction borders ${\mathrm{predictions}=[[X}_{\max}{,X}_{\min}{,Y}_{\max}{,Y}_{\min}{,\mathrm{score}],\;[*],\;[*]]}_{\;}{}_{\;}$ and a given IoU threshold. Output the prediction box result filtered by the NMS algorithm, which is ${[X}_{\max}{,X}_{\min}{,Y}_{\max}{,Y}_{\min},\mathrm{score}]$. NMS simply and directly preserves the prediction box with higher confidence than the maximum threshold. One notable drawback of the NMS algorithm is that when facing the problem of object overlap, the confidence of other objects will be lowered slightly but the prediction box representing another overlapping object will be deleted, seriously affecting the detection of overlapping objects.

**Figure 4:**
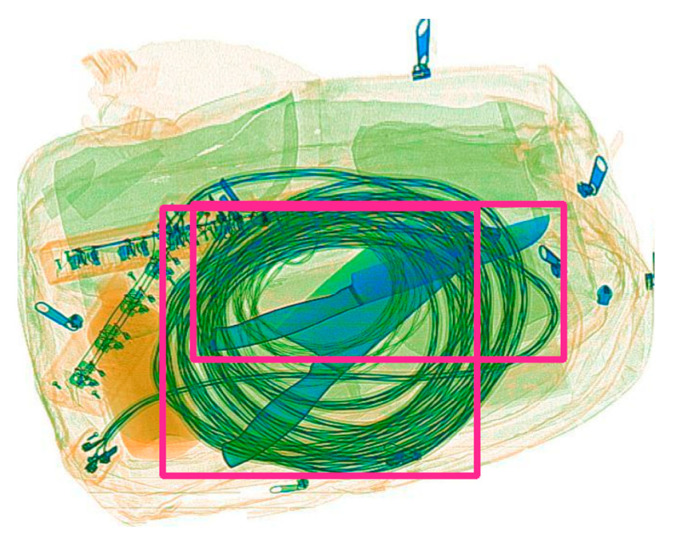
There is a significant overlap in the objects that need to be detected.

The Soft-NMS algorithm does not directly remove the box M with the highest bounding box overlap that is greater than the threshold but reduces its confidence. This method can preserve more boxes and to some extent avoid overlap. As shown in Figure 4, the luggage images captured by X-ray will contain many overlapping items. We will better solve the impact of object overlap by applying Soft-WIoU-NMS in the algorithm.

### 3.6. CBAM

To make the target extraction feature module pay more attention to the fuzzy boundaries of the prohibited area, we use the CBAM module to reassign the feature weights after the first upsampling operation, which we believe is necessary. Given the intermediate feature map, the CBAM module infers the attention map in order with two independent dimensions (channel and space) and then multiplies the attention map and the input feature map to perform adaptive feature optimization.

**Figure 5:**
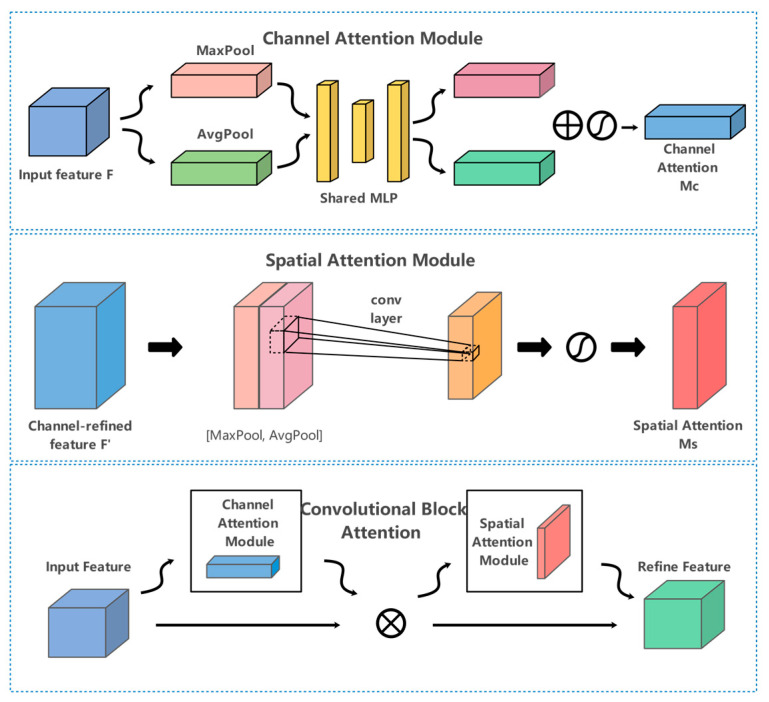
Convolutional block attention module.

Specifically, CBAM is located behind each ELAN module, which can operate with YOLOv7 feature map with different scales, making the network focus more on the foreground and the contextual information in Figure 5.

## 4. Experiment

In this section, we conduct a series of comparative experiments to demonstrate the superiority of our algorithm. We also designed a series of ablation experiments based on the improvements in the attention mechanism, IoU loss function, and NMS.

### 4.1. Experimental Dataset

**Figure 6:**
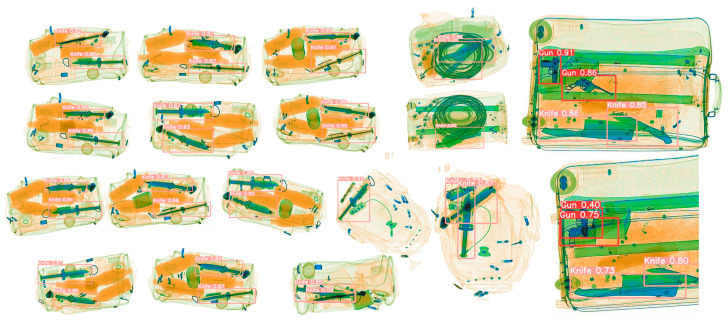
Dataset presentation. There are many overlapping phenomena.

**Figure 7:**
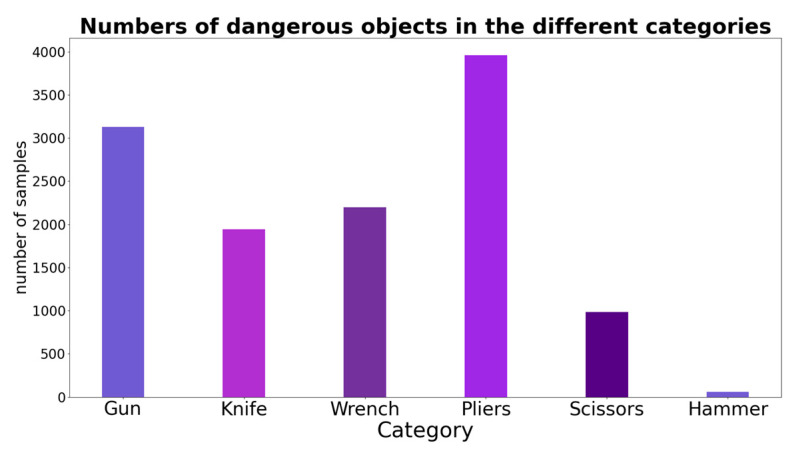
Numbers of dangerous objects in the different categories.

Our model was trained using the public dataset SIXray. This dataset includes 1,059,231 real security inspection photos, of which 8929 are positive samples. The specific dataset and categories can be seen in Figure 6. There are five categories of detection which can be seen on Figure 7: guns, knives, wrenches, pliers, and scissors. The distribution and colours of the objects in the SIXray dataset are basically consistent with reality, with characteristics such as stacking, occlusion, and a cluttered background.

### 4.2. Metrics

Precision refers to the proportion of true positive cases among the samples predicted to be positive and is expressed as *P*. The formula to calculate *P* is shown as follows.

(10) $$P=\frac{\mathrm{TP}}{\mathrm{TP}+\mathrm{FP}}$$

The average precision (AP) is the average precision rate under different recall rates and can expressed by the following formula.

(11) $$\mathrm{AP}=\overset{n}{\underset{i=1}{\sum }}P(i)\Delta r(i)=\int_{0}^{1}p(r)\mathrm{dr}$$

By definition, the mean average precision (mAP) calculates the mean of AP, which is the average precision of all categories. The formula is as follows:

(12) $$\mathrm{mAP}=\frac{\sum_{n=1}^{N}\;\mathrm{AP}(n)}{N}$$

where represents the category and represents the total number of categories.

### 4.3. Comparative Experiments

In this section, we conduct comparative experiments. The experiments were conducted using the PyTorch deep learning architecture and an Ubuntu18.04 system equipped with dual-card NVIDIA 2080Ti 12G. Before the experiment, we divided the 8929 images from SIXray into training and test sets in a 9:1 ratio. To verify the advanced nature of the model, we selected Faster-R-CNN, YOLOv3, YOLOv5, and YOLOv7 for comparison. The comparison results are shown in Table 1.

The selected model is a classic model in the field of object detection that has been the basis for many research improvements and comparative models. This experiment carried out a super parameter comparison on the model, and all of the models used the same learning rate, number of epochs, etc. As shown, our model EM-YOLOv7 combined with Soft-WIoU-NMS has a mAP:95 of 19.7% higher than YOLOv3, 9.9% higher than YOLOv5, 11.8% higher than Fast-RCNN, and 1.1% higher than YOLOv7.

### 4.4. Ablation Study

We designed three sets of controlled trials for ablation experiments, namely, the comparison of detection attention mechanisms, IoU loss function comparison, and Soft NMS comparison under different IoUs. This design can separately detect the enhancement in model performance that results from the three improvements and eliminate their influence on each other.

The comparative experiments indicate that our model improved the accuracy by 1.1% compared to the original model. We speculate that the EM attention module can enhance the feature extraction ability of the images X. We designed a series of attention mechanisms for comparison, hoping to prove the advantages of the EM attention module for item detection in X-ray images. Therefore, we used classic attention SE and CBAM for comparison. The results (Table 2) indicate that SE and CBAM do not perform well with X-ray images, and EM attention is 1% higher. After adding the backbone to CBAM, a comparison was made with SE at the same location, and it was found that SE had poor performance.

We evaluated the detection performance of three different versions: SIoU, DIoU, and WIoU. This allows us to select the IoU loss function that is more suitable for X-ray datasets while excluding the impact of the other modules. As shown in the table, the accuracy of the Wiouv1 version is 0.6%, 0.7%, 0.6%, and 0.4% higher than that of the other IoU versions as shown in Table 3, our model with WIoUv1 has good performance.

Finally, we designed experiments to evaluate the performance of Soft NMS under different IoU loss functions. Under YOLOv7(base) and WIoU, as shown in Table 4, the training results with Soft NMS are 0.2% and 0.3% higher than before. However, the accuracy in the gun and wrench categories decreased by approximately 0.5%, but the detection performance in the other categories was better. SIoU and DioU are not suitable for linking to Soft NMS, as the performance decreased by approximately 1%.

### 4.5. Analysis of the Results

From the comparative experimental results, it can be seen that our EM-YOLOv7 model has an improvement in accuracy of 1.1% compared to the SOTA YOLOv7 model. In contrast, classic models such as YOLOv3, YOLOv5, and Faster RCNN have not achieved suitable performance for deployment due to outdated trips and insufficient adaptability to special tasks.

On the basis of the comparative experiment results, we designed ablation experiments to evaluate the effectiveness of our designed EM-Attention, IoU, and Soft NMS. In the first ablation experiment on attention mechanism, our EM-Attention has a 0.3% higher accuracy than YOLOv7(base). In addition, it can be seen from the experimental data that SE attention mechanisms does not perform well on X-ray images. In the second experiment, we found that the performance of WioUv1 is more suitable for SIXray data. Although this version is 0.1% more effective than YOLOv7-base (CIoU), subsequent experiments have shown that Wiouv1 is more suitable for use with SoftNMS. In the third experiment on Soft NMS, it was demonstrated that, firstly, YOLOv7(base) with Soft NMS exhibited 0.2% higher accuracy, and secondly, Soft-WIoU-NMS had better performance. Although there are a few categories in which the accuracy was slightly lower, after analysis we believe that these decreases are due to experimental errors or insufficient overlap performance of these categories in the dataset. For example, items with multiple overlaps, such as knives, have significantly improved detection accuracy.

## 5. Conclusions

In this paper, we study the detection of prohibited items in X-ray inspection images, which is a detection field with unique image characteristics. We found that researchers have added modules that are more suitable for X-ray image features, such as SSD and YOLOv5, to a series of mature detectors. However, the selected detector is not SOTA, and the basic performance of the detector is flawed. To facilitate research in this field, we used a high-quality YOLOv7 model as the benchmark with most practical X-ray dataset (SIXray) images as the training dataset. To overcome the issues of background clutter and object overlap in X-ray image detection, we propose the edge material attention module (EM-Att), which is used in the preprocessing stage of the image input backbone network. This module can extract features based on the features of X-ray images and uses the latest detection model YOLOv7. We use Soft WIoU NMS to solve the problem of object overlap during the detection process and add the CBAM attention mechanism to the backbone to extract features. It has been experimentally proven that our module can improve the performance of the most advanced detection methods, significantly outperforming several widely used attention mechanisms. This module is suitable for deployment in the real world to assist with manual detection.

## Figures and Tables

**Table 1:** Comparison of different detection algorithms on SIXray.

Method	mAP50	mAP50:95	Category				
			Gun	Knife	Wrench	Pliers	Scissors
YOLOv3	0.876	0.545	0.688	0.382	0.466	0.583	0.605
YOLOv5	0.91	0.643	0.737	0.553	0.614	0.681	0.631
Fast-RCNN	0.903	0.624	0.718	0.522	0.596	0.652	0.634
YOLOv7	0.951	0.731	0.783	0.671	0.732	0.743	0.726
EM-YOLOv7 (Ours)	0.958	0.742	0.789	0.683	0.751	0.757	0.731

**Table 2:** Comparison of the ablation experiments on different attention mechanisms.

Method	mAP50	mAP50:95	Category				
			Gun	Knife	Wrench	Pliers	Scissors
EM-YOLOv7	0.951	0.731	0.783	0.671	0.732	0.743	0.726
EM-YOLOv7 + SE	0.946	0.725	0.779	0.666	0.723	0.737	0.718
EM-YOLOv7 + CBAM	0.958	0.742	0.789	0.683	0.751	0.757	0.731

**Table 3:** Results of ablation experiments with different IoU loss.

Method	mAP50	mAP50:95	Category				
			Gun	Knife	Wrench	Pliers	Scissors
YOLOv7(base)	0.951	0.731	0.783	0.671	0.732	0.743	0.726
YOLOv7 + SIOU	0.948	0.724	0.782	0.661	0.704	0.748	0.725
YOLOv7 + DIOU	0.95	0.723	0.771	0.664	0.717	0.741	0.723
YOLOv7 + WIOUv1	0.952	0.73	0.777	0.667	0.731	0.745	0.731
YOLOv7 + WIOUv2	0.95	0.724	0.774	0.659	0.721	0.745	0.718
YOLOv7 + WIOUv3	0.948	0.726	0.779	0.668	0.714	0.745	0.723

**Table 4:** Results of ablation experiments with different IoU loss and Soft NMS.

Method	mAP50	mAP50:95	Category				
			Gun	Knife	Wrench	Pliers	Scissors
YOLOv7(base) + Soft-NMS	0.952	0.733	0.778	0.674	0.741	0.745	0.729
YOLOv7 + Soft-NMS (SIoU)	0.943	0.72	0.762	0.663	0.699	0.748	0.727
YOLOv7 + Soft-NMS (DIoU)	0.936	0.725	0.778	0.677	0.71	0.736	0.723
YOLOv7 + Soft-NMS (WIoU)	0.955	0.734	0.785	0.68	0.733	0.743	0.731

## Data Availability

Not applicable.
